# Macroalgae of Izmir Gulf: *Cystoseira barbata*, *Cystoseira compressa* and *Cystoseira crinita* species have high α-glucosidase and Moderate Pancreatic Lipase Inhibition Activities

**DOI:** 10.22037/ijpr.2020.1100953

**Published:** 2020

**Authors:** Fatma Gül Çelenk, Atakan Sukatar

**Affiliations:** a *Department of Medical Genetics, Faculty of Medicine, Ege University, Izmir, 35040, Turkey. *; b *Department of Biology, Faculty of Science, Ege University, Izmir, 35040, Turkey.*

**Keywords:** Inhibitors, Alpha-glucosidase, Pancreatic lipase, Cystoseira, HT29

## Abstract

Hyperglycemia and hyperlipidemia have been symptoms of many serious diseases such as diabetes and atherosclerosis overall the world. Thus, drug researchers have focused on new, natural and healthy drug alternatives. Marine macroalgae is a great source of hypoglycemic, hypolipidemic or hypocholesterolemic agents. In this study, we investigated that hypoglycemic, hypolipidemic and cytotoxic potentials of 22 marine macroalgae from the Gulf of Izmir. According to our results, the cold methanol extract of *Polysiphonia denudata* exhibited the highest antioxidant activity (93.6%) compared to BHA (95.3%). Three *Cystoseira* species, *Cystoseria crinita* (91.9%), *Cystoseria barbata* (90.7%), *Cystoseria compressa* (89.8%) showed higher α-glucosidase inhibition rates than oral antidiabetic acarbose (79.5%). It has also been observed that same species are potent inhibitors of pancreatic lipase. Cytotoxicity test revealed that these extracts did not cause viability inhibition on MCF-7. The results of maltose- glucose assay indirectly displayed that *Cystoseira* cold methanolic extracts inhibited maltose consumption better than acarbose on HT29. The results of this screening study show that these *Cystoseira *species may provide non- toxic bioactive agents to control non-communicable diseases (NCDs) such as cardiovascular disease and diabetes mellitus.

## Introduction

International Diabetes Federation (IDF) has published in IDF Diabetes Atlas – 7^th^ editions that there are 415 million adults aged 20-79 with diabetes worldwide and a further 318 million adults are estimated to have impaired glucose tolerance. World Health Organization (WHO) has noticed that among the non- communicable diseases, cardiovascular diseases (CVDs) are the number one cause of death. 

Diabetes mellitus (DM) is classified Type 1 DM, Type 2 DM and gestational DM. Type 1 DM is certain insulin deficiency and is treated with insulin preparations. Type 2 DM is associated with insulin resistance in which cells fail to respond to insulin and main goal of its treatment is to keep stable blood glucose level via glucosidase inhibitors such as acarbose, miglitol and voglitol (1)usually leading to absolute insulin deficiency. The powerful synthetic α-glucosidase and α-amylase inhibitors such as acarbose, voglitol and miglitol cause sharp decrease in blood glucose level (2). Therefore, the main approach to alleviate diabetes is to inhibit the enzymes that hydrolase carbohydrates, and by this way, create a retardation to glucose absorption (3, 4). Because the higher glucose concentrations in blood cause higher ROS productions in endothelial cells via mitochondrial electron transport chain and advanced glycation end products (AGEs) (5). Then ROS activates NFκB and this leads to inflammatory response (including cytokines, acute phase reactants and cell adhesion molecules) and vasomotor imbalance. ROS resulting hyperglycemia could lead to lipid peroxidation, which yields advanced lipoxidation end products (ALEs) (6). As a result a cardiovascular disease might occur, such as atherosclerosis (7). CVD is more complex in both diagnosis and treatment. The preliminary treatment of CVD is focused on diet and life style interventions (8). Then, lipase inhibitors, such as orlistat, are used to decrease gastrointestinal absorption of lipids. By this way, elevated lipid levels in blood is decreased (9). On the other hand, it is commonly known the adverse effects of these kinds of synthetic agents for long- term use, such as flatulence, abdominal discomfort, kidney disease (2, 10 and 11) Natural antioxidants can attenuate these damaging effects of ROS related diabetic cardiovascular complications (12–14)

It is evident that macroalgae provide a great source for definable crude natural products which have less adverse effects and toxicity than their synthetic equivalents (15–18). Fucoidans, phycocolloides and phenolic compounds produced by brown macroalgae exhibit α-glucosidase inhibition potentials (19–22). Some of the macroalgal polysaccharides and fibers such as alginates, carrageenan, fumaran, laminaran, porfiran and ulvan, cause hyperlipidemia and hypoglycemia (21, 23–26) Researches have suggested different mechanism of action for macroalgal substances: 1) Enzyme inhibition studies for glucosidases and/or lipase are used to determine inhibitor potential of individual crude extract or identified compound (19, 25 and 27–31), 2) Responses of cells to high glucose concentration or glucose uptake assays, or lipid accumulation in adipocytes demonstrate the protective effects (22, 29 and 32), 3) oral or intraperitoneal administrations of macroalgal substances to experimental animals give information about vital effects on insulin, plasma glucose and fatty acid levels in blood (20, 23 and 33–36). These parameters help investigate the effects of macroalgal substances on hyperglycemia, postprandial hyperglycemia, hyperinsulinemia or hyperlipidemia. 

## Experimental

*Collection of Macroalgal Material*

Marine macroalgal species were collected from coastlines of Izmir Gulf. All macroalgal specimens belong to Chlorophyta, Rhodophyta and Phaeophyceae were placed in the labelled plastic bags (approximately 0.5-1 kg wet weight) and immediately transported to the laboratory in cooled containers. To remove sand and epiphytes, algal thalli were gently rinsed in seawater. Taxonomic identification was done in terms of their morphology. Specimens had been stored at -20 °C until experiments begun. The voucher specimens are preserved in the Herbarium of Ege University.

*Preparation of Macroalgal Extracts*

Wet tissues from each species were lyophilized. One gram of powder was extracted with 10 mL of methanol (Labscan, TH) by shaking in an orbital shaker at 300 rpm overnight at +4 °C. Extracts were then centrifuged at 4000 rpm for 10 min and supernatants were collected. Collected supernatants were placed in orbital shaker and extraction was initiated again. This procedure had been repeated for 10 days. After 10 days of extraction/centrifugation cycle, the collected solvent was removed by rotary evaporation and powders of the crude extracts were dissolved in methanol for Thin Layer Chromatography (1 mg mL^-1^) and DMSO (dimethyl sulfoxide, Sigma-Aldrich, USA; 10 mg crude extract per mL) for further experiments. All sample stored at -20 °C. 

*Thin Layer Chromatography*

Extraction efficiency was roughly evaluated by thin layer chromatography. Crude macroalgal methanol extracts were dissolved in methanol (1 mg mL^-1^). Individual samples were loaded four times on silica coated aluminum TLC (Merck, DE) plate as a dash. The solvent system was chloroform: ethyl acetate: methanol (5: 5: 1, v/v; Sigma-Aldrich, USA, Riedel- del Hansen, DE; Labscan, TH, respectively). As the solvent slowly migrated up the plate, the components of the extract migrated up at different rates. Individual extracts were separated into different coloured dashes. The TLC profiles of plates were visualized after dipping into hydrochloric acid/methanol (1: 9, v/v; Sigma-Aldrich, USA; Merck, DE) and heating until getting dark. All the coloured or invisible dashes were observed as dark dashes on plates. 

*Antioxidant Assay with β-carotene/linoleic acid system*

The β-carotene/linoleic acid activities of samples were evaluated by β-caroten bleaching system with the minor modifications for 96-well plate (37)gallic acid (GA. Eight microliter of linoleic acid (Sigma-Aldrich, USA) were pipetted into eppendorf containing 800 µl of β-carotene (Sigma-Aldrich, USA; 1 mg mL^-1^) in chloroform (Sigma-Aldrich, USA; 1 mg mL^-1^) and added 80 µL of Tween 40 (Sigma-Aldrich, USA). The chloroform was blown away by nitrogen flow. This mixture was pipetted slowly in 20 mL of distilled water with vigorous agitation to form an emulsion. Twenty microliter of blank (ethanol), positive control (BHA: Buthyl-4-hydroxyanisole, 30 mg mL^-1^) or samples (5 mg mL^-1^) were loaded 96-well plate and added 200 µL of the emulsion and the absorbance was measured immediately at 450 nm. The plate was placed in a dark room for 90 min. The absorbance was measured again. All measurements were carried out in triplicate. The antioxidant activity of extracts was evaluated using the following Equation: 

AA (%) = [1 - (A_0 _- A_90_)/(A’_0 _- A’_90_)] **×** 100

Where the A_0_ and A’_0_ are the absorbance values measured at zero time of the sample and the control, respectively, and A_90_ and A’_90_ are the absorbance values measured at 90 min of the sample and the control. 

*Cell Culture and Microscopy*

All studied cell lines were kindly provided by Ege University Medical School Department of Medical Oncology. Breast cancer cell line MCF-7 was cultured in RPMI medium (Lonza, CH) with 10% fetal bovine serum (Biological Industries, USA) and penicillin-streptomycin (100 U mL^-1^; Sigma-Aldrich, USA) supplement. Cultures were maintained in CO_2_ incubator with standard incubation conditions. Cells were treated with different concentrations of macroalgal methanol extracts reconstituted in dimethyl sulphoxide (DMSO; Sigma-Aldrich, USA) and diluted with proper media for viability experiments. For each experimental procedure, appropriate concentration of vehicle (DMSO) was used as a carrier control. Human colon adenocarcinoma cell line HT29 was cultured in McCoy’s 5A Medium (Biochrom, UK) with 10% fetal bovine serum (Biological Industries, USA), penicillin-streptomycin (100 U mL^-1^; Sigma-Aldrich, USA) and 1% L-glutamine (Gibco, USA) supplement. Cultures were maintained in CO_2_ incubator with standard incubation conditions in 96-well plates.

*Enzyme Inhibition Tests*

*α-glucosidase enzyme inhibition test*

The enzyme inhibition activity of extracts for α-glucosidase was assessed using the PNG (4-nitrophenil-α-D-glucopyranoside; Sigma-Aldrich, USA) as substrate and *Saccharomyces cerevisiae* α-glucosidase as enzyme (Sigma-Aldrich, USA) with 96-well plates. Fifty microliter of PBS (100 mM; pH 7.5) were loaded into wells and 2 µL of samples (1 mg mL^-1^), acarbose (250 mg mL^-1^; as positive control) or PBS (for enzyme reaction) were added. Fifteen microliter of α-glucosidase was added, except for blank. The plate was pre- incubated at 37 °C for 10 min. Fifteen microliter of PNG (3 mM) was added into wells and the plate was incubated at 37 °C for 30 min. The enzymatic hydrolysis of substrate was monitored by the amount of ρ-nitro phenol released in the reaction mixture at 410 nm where the enzymes were replaced buffer. The inhibition percentage of α-glucosidase was assessed by following Equation: 

Inhibition (%) = [1 - (A_e _- A_s_)] × 100

Where the Ae is the absorbance value of enzyme reaction and A_s_ is the absorbance value of extract added reaction.

*Pancreatic lipase enzyme inhibition test*

Pancreatic lipase enzyme inhibition test was designed as fluorometric assay for 96-well plates (black flat bottom). Fifty microliter phosphate buffer with CaCl_2_ (Ca-PBS; 0.1 mM CaCl_2_, pH 7.5), 50 µL of pancreatic lipase (Sigma-Aldrich, USA; 2 mg mL^-1^) were loaded into wells. Twenty microliter of sample (5 µg mL^-1^), orlistate (Sigma-Aldrich, USA; positive control, 15 mg mL^-1^) or Ca-PBS (for enzyme reaction) were added and then the plate was pre-incubated at 37 °C for 10 min. After the pre-incubation, 100 µL of 4-methylumbelliferyl oleate (4-MU; Sigma-Aldrich, USA; 0.1 mM) was pipetted into wells and the plate was incubated 37 °C for 30 min. The amount of 4-MU released by the lipase measured using a fluorescence spectrometer at an excitation wavelength of 320 nm and an emission wavelength of 450 nm. The inhibition percentage of pancreatic lipase was assessed by following Equation: 

Inhibition (%) = 100 - (A_s_ × 100)/A_c_

Where A_s_ is the absorbance value of extract added reaction, A_c_ is the absorbance value of enzyme reaction.

*Inhibition of Cell Viability*

Growth inhibitory effects of extracts were investigated via mitochondrial dehydrogenase activity by WST-8 colorimetric assay kit (Sigma-Aldrich, USA). Briefly, MCF7 cells were seeded in 96-well plate 10 × 103 cells per well and incubated overnight for both cell attachment and growth. Macroalgal extracts were added to wells in different final concentrations (0, 1, 5, 10, 25, 50 and 100 µg mL^-1^) with six repeats. Maximum DMSO concentration was 1%. After 72 h of incubation, 10 μL of WST-8 reagent was added to wells and plates were incubated at 37 °C for 15 min for the formation of color. Absorbance was measured at 450 nm on multi-well spectrophotometer. Untreated cell viability was considered as 100% and extract-treated cell viabilities were calculated accordingly. IC_50_ (inhibitory concentration 50) values were calculated using CalcuSyn v2.0.

*Determining of Maltose Consumption*

α-glucosidases is a glucosidase which breaks down the 1,4-α bonds in starch or disaccharides to produces glucose (38) Maltose disaccharide was used as sole carbon source in experimental media to observe α-glucosidase activity and effects of extracts on it. Maltose concentrations were adjusted to glucose concentration of McCoy’s 5A medium. Acarbose was positive control for inhibition. The decrease of maltose level in experimental media showed us maltose consumption, indirectly. Stable maltose levels were considered as indicator for inhibition activity of acarbose or extracts. 

After confluence of HT29 cells, McCoy’s 5A- based media was removed and wells were gently washed with PBS, then 200 µL of three experimental media were added to the wells, individually: (i) PBS with maltose (28 mM), (ii) PBS with maltose (28 mM) and acarbose (250 mg mL^-1^), (iii) PBS with maltose (28 mM) and extract (10 mg mL^-1^). After 37 °C for 6 h incubation period, 100 µL of experimental media were transferred clean Eppendorf tubes. Maltose- Glucose Assay Kit (Abcam, UK) was used to determine and calculate the decrease of maltose concentrations. 

*Statistical Analysis*

Standard deviations of enzyme inhibition tests were calculated using Microsoft Office Excel and IC_50_ values were determined by CalcuSyn 2.0 software*.*

## Results

Samples of 22 macroalgae belonging to Chlorophyta, Rhodophyta and Phaeophyceae collected from İzmir Gulf were listed Table 1. Their collection dates, collection places and coordinates were published in our previous study (39).

**Figure 1:**
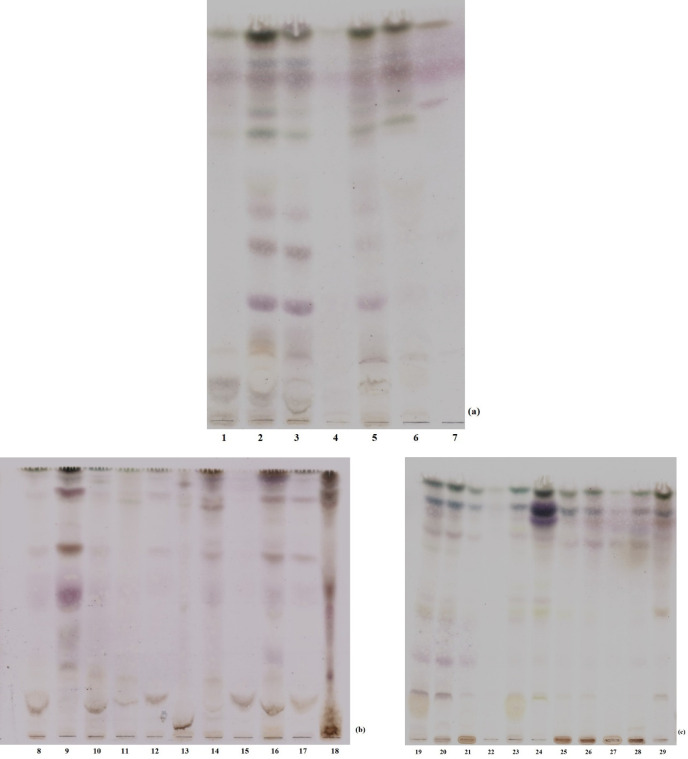
TLC profiles of macroalgae. Some of the macroalgal extracts were excluded from study (3, 4, 12, 17, 20, 22 and 25). (a) The members of Chlorophyta: (1) *Cladophora sp*., (2) *Ulva linza*, (5) *Ulva rigida*, (6) *Chaetomorpha aerea*, (7) *Codium fragile*. (b) The members of Rhodophyta: (8) *Gracillaria gracilis*, (9) *Polysiphonia denudata*, (10) *Osmundea pinnatifida*, (11) *Gelidium pulchellum*, (13) *Acanthophora nayadiformis*, (14) *Ceramium virgatum*, (15) *Gelidium spinosum*, (16) *Laurencia obtusa*, (18) *Palisada perforata*. (c) The members of Phaeophyceae: (19) *Petalonia fascia*, (21) *Colpamenia sinuosa*, (23) *Halopteris filicina*, (24) *Dictyota dichotoma*, (26) *Cystoseira compressa*, (27) *Cystoseira crinita*, (28) *Cystoseira barbata*, (29) *Dictyota spiralis*

The TLC fingerprint profiles of macroalgal extracts in chloroform: ethyl acetate: methanol solvent system was presented in terms of their taxonomic status in Figure 1. The retardation factor (Rf) values were not calculated, because the test set is composed of the crude extracts. Visible bands have shown that cold methanol is suitable solvent for extraction of seaweeds. 

Inhibition activities of macroalgal extracts for β- carotene/linoleic acid bleaching were presented in Table 1. *Polysiphonia denudata *(Dillwyn) Greville ex Harvey (EGE41707), a member of Phaeophyceae, exhibited the highest inhibition percentage (93.6%). The highest average antioxidant activity was calculated for Chlorophyta (52.1%). *Ulva linza* Linnaeus (EGE41706), with the 91.4%, showed the highest antioxidant activity. Other species belong to Chlorophyta exhibited lower activities, and this situation cause decrease in the average activity of Chlorophyta. Rhodophyta (46.3%) and Phaeophyceae (40.4%) follow it. Except for *Polysiphonia denudate (P. denudata)* and *Ulva linza (U. linza)*, it is observed that all macroalgal species have moderate antioxidant activities when compared to positive control BHA (95.3%). 

The α-glucosidase inhibitory activities of the methanol extracts were shown in Table 1 as percentage inhibitions. The inhibition rates were ranged between 0.0–91.9%. The members of Rhodophyta exhibited lower inhibition average (8.9%) than those of Chlorophyta (32.6%). However, the members of Phaeophyceae displayed stronger inhibitor effects (39.4%). Especially, three *Cystoseira* species, *Cystoseria crinita* Duby (EGE41722; 91.9%),* Cystoseria barbata* (Stackhouse) C. Agardh (EGE41720; 90.7%), *Cystoseria compressa* (Esper) Gerloff and Nizamudd (EGE41721; 89.8%) showed quite higher α-glucosidase inhibition rates than positive control acarbose (79.5%). 

The pancreatic lipase inhibition percentages of the methanol extracts are seen in Table 1. Orlistat was used as positive control (95.2%). However, *Cystoseira compressa* *(C. compressa) *displayed lower pancreatic lipase inhibitory activity than orlistate (79.5%); it showed the highest pancreatic lipase inhibitory activity among macroalgal specimens. The zero inhibition rates were exhibited by both *P. denudata* and *Gelidium spinosum *(S.G. Gmelin) P.C. Silva (EGE41717). Interestingly, the members of Phaeophyceae*, *which showed the higher α-glucosidase inhibitory activities also*,* showed the higher pancreatic lipase inhibition rates: *C. compressa* (79.5%), *Cystoseira barbata *(*C. barbata*; 73.8%), *Cystoseira crinita *(*C. crinite*; 61.5%). In addition to them, three members of Chlorophyta, *Chaetomorpha aerea* (Dillwyn) Kützing (EGE41703), *Ulva rigida* C. Agardh (EGE41705) and *U. linza* also had the higher inhibitory rates for pancreatic lipase; 71.1, 70.5 and 69.3%, respectively. 

**Figure 2:**
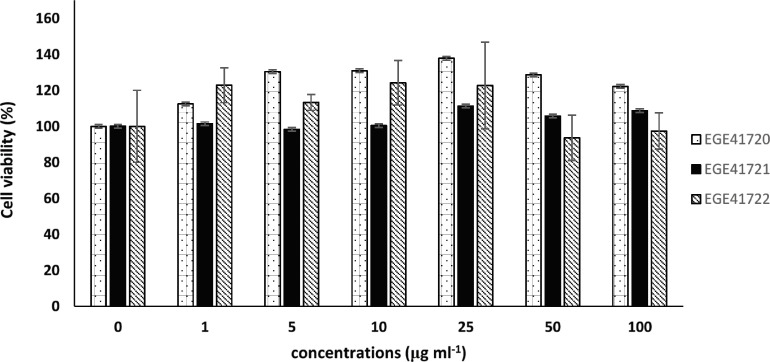
*Cystoseira* species do not inhibit the cell viability at the administered doses. EGE41721: *C. compressa*, EGE41722: *C. crinita*, EGE41720: *C. barbata*

Three members of Phaeophyceae, which exhibited good α-glucosidase and pancreatic lipase enzyme inhibition activity, were preferred to determine their cytotoxic activity. These macroalgal species did not exhibit a viability inhibition on MCF-7 at the administered doses (Figure 2). 

**Figure 3:**
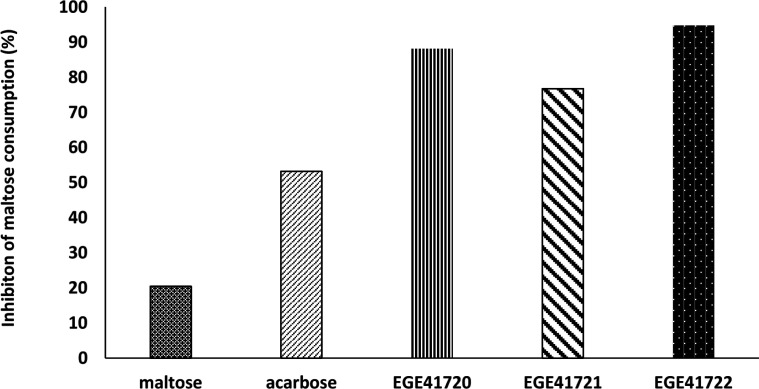
*Cystoseira* species exhibit stronger inhibition than acarbose on α-glucosidase enzyme of HT29 at the administered doses. EGE41721: *C. compressa*, EGE41722: *C. crinita*, EGE41720: *C. barbata*

Post- incubation maltose concentrations of experimental media were measured using maltose- glucose assay kit. The decrease of maltose level was considered as an indirect sign for inhibiton of α-glucosidase enzyme of HT29 cells. Although, the inhibition percentages was variable, the cold methanol extracts of *Cystoseira* species, *C. crinita*, *C. barbata* and *C. compressa*, were effective on maltose consumption. The results showed that these *Cystoseira* species exhibited strong inhibition on α-glucosidase enzyme of HT29 cells (94.8%, 88.2% and 76.1%, respectively) compared to positive control acarbose (53.2%, Figure 3).

## Discussion

Diabetes mellitus and cardiovascular diseases affect about millions of adults overall the world. The factors, such as mortality rates, drug resistance and adverse effects of medicines, prompt exploration for new agents. In this study, as a second part of our screening study, we have evaluated the antioxidant hypoglycemic and hypolipidemic activities of marine macroalgae collected from Izmir Gulf. 

The first results of this screening study showed that collected macroalgae from this region displayed antioxidant and anticancer activities (39). The results revealed that *P. denudata *had exhibited strong 2, 2-diphenyl-1-picryl-hydrazyl inhibition activity (DPPH; 86.7%) and high total phenolic content (TPC; 245.9 mg GAD). Current study provides a crosscheck to show strong antioxidant capacity of *P. denudata *in β-caroten/linoleic acid system. It has also been reported that *P. denudata* displayed moderate cell viability inhibition on MCF7 cells (39) and *Artemis salina *(40). There has been no data about hypoglycemic or hypolipidemic activity of *P. denudata* in literature, until today. Our findings are the first records that adduce the hypoglycemic and hypolipidemic potential of *P. denudata*. However, the solvent types for extraction and preferred organisms for viability test are variable; the researchers have noticed high antioxidant activity and moderate cytotoxicity of *P. denudata *(39, 41 and 42)*. *

*Cystoseira* genus consists of 293 species (and infraspecific), of which 46 have been flagged as currently accepted taxonomically and *Cystoseira* genus was represented by three species in our study (42). It had been observed that they have variable antioxidant activity in DPPH system and total phenolic content. Our recent results also substantiated their variable antioxidant activities via β- carotene/linoleic acid system. It was reported that the hydroquinons isolated from *C. crinita* have high antioxidant activity and its meroterpenoids exhibit weak cytotoxicity on HM02, HEPG2 and MCF7 cells (43). According to our results based on MCF-7 cytotoxicity of *C. compressa*, the former findings proved that its methanol extract showed very low cell viability inhibition on HEP3B cells (44). The crude methanol extract of *C. barbata* exhibited lower antioxidant activities; however, it has been indicated that its fucan and fucoidan displayed higher antioxidant activities (45, 46) . In addition, it has been reported that the fucoidans isolated from *C. crinita* and *C. compresssa* showed similar anti-radical, anti-inflammatory and gastroprotective effects and their aqueous extracts displayed anti-proliferative effects on cell lines and anti-inflammatory effects on male adult Wistar rats (47, 48)Cystoseira compressa and Cystoseira crinita. 

Researchers suggested that the techniques used for determining DPPH scavenging activity, reducing activity, and β-carotene-linoleic acid system assays present simple, fast, and reliable evidence to assessment of the total antioxidant activity of the extracts (49). Their finding of a significant correlation between the DPPH radicals scavenging activities and the reducing activities were explained by the fact that both assays rely on electron/hydrogen donation. On the other hand, the researchers reported that the emulsified lipid used in the β-carotene -linoleic assay introduced additional variables that affect the oxidation process in comparison to the other methods. In terms of our former findings, there was significant correlation between DPPH scavenging activity and TPC, and Phaeophyceae members displayed higher antioxidant activity. Nevertheless, our last results obtained by β-carotene-linoleic assay showed that Chlorophyta features the highest antioxidant activity potential than Phaeophyceae and Rhodophyta (52.1, 46.3 and 40.4%, respectively).

The extracts of *Cystoseira* members have generally distinguished their enzyme inhibiton capabilities (50–54). We observed that both α-glucosidase and pancreas lipase enzyme inhibition assays showed that their extracts might include strong inhibitor molecules. It has shown that *C. crinita* is a source of sulphated polysaccharides which inhibit the key enzymes of diabetes and hypertension (51). These researchers have reported that the administration of *C. crinita* sulphated polysaccharides protects pancreas β- cells from death and damage and attenuates α- amylase activity in serum, pancreas and small intestine in diabetic rats. They suggested that the decrease in α- amylase level and increase in insulin level lead to a decrease in glucose rate. 

Our screening study revealed that three members of Chlorophyta have good inhibitory rates for pancreatic lipase compared to orlistat. Cold methanol extract of *Chaetomorpha aerea* *(C. aerea*) may probably include inhibitor compounds for pancreatic lipase. However, it did not inhibit α-glucosidase enzyme. Similarly, recent results in literature, the chloroform extract of *C. aerea* did not show effective inhibition against α- glucosidase, but it was reported that when used alpha-amylase, instead of this enzyme, the extracts exhibited good inhibitor activity (55). There is no record for hypolipidemic activity of this species. *Ulva rigida (U. rigida*) exhibited hypoglycemic activity on a certain level, but its hypolipidemic activity was slightly higher than this. *In-vivo* study conducted with streptozotocin- induced diabetic rats revealed that ethanol extract of *U. rigida *showed both antidiabetic and antihyperlipidemic effects and improves carbohydrate metabolism (56). 

This screening research stated the antioxidant, hypoglycemic and hypolipidemic activities of marine macroalgae of İzmir Gulf *in-vitro*. Based on the results, with the certain levels of tested biological activities, these macroalgal species appear as good producers of bioactive molecules. Hence, further isolation and identification studies and *in-vivo *studies based on these bioactive molecules are going to be our next step to lead to evaluate for medical potential.

**Table 1:** Antioxidant activity and enzyme inhibition effects of macroalgae

**Family**	**Voucher** **ID**	**Species**	**Antioxidant Activity (%)**	**α-glucosidase inhibition** **(%)**	**Pancreatic** **lipase ihibition** **(%)**
*Ulvaceae*	EGE41706	*Ulva linza* Linnaeus	91.4	5.8 ± 0.82	69.3 ± 3.0
	EGE41705	*Ulva rigida* C. Agardh	15.2	54.6 ± 0.10	70.5 ± 4.74
*Codiaceae*	EGE41711	*Codium fragile* (Suringar) Hariot	44.3	24.5 ± 0.05	14.4 ± 0.7
*Cladophoraceae*	EGE41703	*Chaetomorpha aerea* (Dillwyn) Kützing	68.5	12.5 ± 1.11	71.1 ± 2.3
	EGE41700	*Cladophora* sp.	41.2	13.3 ± 0.10	40.6 ± 9.2
*Rhodomelaceae*	EGE41704	*Osmundea pinnatifida* (Hudson) Stackhouse	55.5	2.2 ± 1.42	45.3 ± 4.0
	EGE41726	*Laurencia obtusa* (Hudson) J. V. Lamouroux	50.5	10.7 ± 0.06	57.2 ± 2.9
	EGE41725	*Palisada perforata* (Bory de Saint- Vincent) K. W. Nam	35.5	0 ± 0.09	55.9 ± 1.8
	EGE41707	*Polysiphonia denudata* (Dillwyn) Greville ex Harvey	93.6	10.1 ± 0.27	0 ± 2.0
	EGE41712	*Acanthophora nayadiformis* (Delile) Papenfuss	37.7	31.5 ± 0.79	20.2 ± 2.5
*Gracilariaceae*	EGE41701	*Gracilaria gracilis* (Stackhouse) M.Steentoft, L. M.Irvine and W. F. Farnham	22.3	1.1 ± 1.94	42.1 ± 4.8
*Ceramiaceae*	EGE41716	*Ceramium virgatum* Roth	54.6	6.2 ± 0.58	44.4 ± 6.1
*Gelidiaceae*	EGE41717	*Gelidium spinosum* (S. G. Gmelin) P. C. Silva	6.8	11.0 ± 1.32	0 ± 1.0
	EGE41702	*Gelidium pulchellum* (Turner) Kützing	59.8	7.7 ± 1.66	24.7 ± 12.0
*Scytosyphonaceae*	EGE41709	*Petalonia fascia* (O. F. Müller) Kuntze	52.6	13.9 ± 0.10	46.2 ± 2.38
	EGE41833	*Colpomenia sinuosa* (Mertens ex Roth) Derbès and Solier	9.0	8.6 ± 0.00	5.9 ± 1.0
	EGE41708	*Dictyota spiralis* Montagne	20.0	0 ± 0.36	23.8 ± 8.8
	EGE41715	*Dictyota dichotoma* (Hudson) J. V. Lamouroux	56.3	3.7 ± 0.19	58.4 ± 22.5
*Stypocaulaceae*	EGE41718	*Halopteris flicina* (Grateloup) Kützing	27.6	16.2 ± 0.70	36.3 ± 11.0
*Sargassaceae*	EGE41719	*Cystoseria barbata* (Stackhouse) C. Agardh	58.7	90.7 ± 0.01	73.8 ± 2.17
	EGE41721	*Cystoseria compressa* (Esper) Gerloff and Nizamudd	53.5	89.8 ± 0.02	79.5 ± 2.9
	EGE41722	*Cystoseria crinita* Duby	45.3	91.9 ± 0.00	61.5 ± 6.0
BHA	-	-	95.3	-	-
Acarbose	-	-	-	79.5 ± 0.07	-
Orlistat	-	-	-	-	95.2
